# Pattern electroretinography response in amblyopic adults

**DOI:** 10.1007/s10792-024-03042-8

**Published:** 2024-02-16

**Authors:** Andresa Fernandes, Nuno Pinto, Ana Rita Tuna, Francisco Miguel Brardo, Maria Vaz Pato

**Affiliations:** 1 Faculty of Health Sciences, University of Beira Interior, Covilhã, Portugal; 2 Faculty of Health Sciences, Rua Marquês de Ávila E Bolama, CICS-Health Sciences Research Centre, University of Beira Interior, 6201-001 Covilhã, Portugal; 3 Department of Physics, University of Beira Interior, Covilhã, Portugal

**Keywords:** Amblyopia, Pattern electroretinography, Retinal disorders, Adults, Electrophysiology

## Abstract

**Introduction:**

Amblyopia is generally a unilateral disorder, defined by at least a difference of two lines of visual acuity between both eyes with the best-corrected visual acuity, a decrease in contrast sensitivity, and a decrease in stereopsis. Pattern electroretinogram (PERG) is a noninvasive technique that provides a retinal biopotential and is a highly sensitive indicator of changes in the macular area. Our aim was to evaluate if there are differences in the retinal response of an amblyopic eye compared with a normal eye (NE).

**Methods:**

We evaluated twenty-four adult volunteers, twelve amblyopes (mean 43.42 ± 12.72 years old), and twelve subjects with NE (mean 35.58 ± 12.85 years old). None of the subjects in the two groups had comorbidities. A complete optometric examination was performed including parameters such as visual acuity (VA) by far and near with ETDRS chart, eye alignment with cover test, and evaluation of retinal cells response with PERG.

**Results:**

The refractive error found in the NE group of subjects had a mean of − 0.95 ± 1.65D, while the amblyopic group showed a mean of − 2.03 ± 4.29D. The VA in amblyopic eyes had a mean of 0.38 ± 0.20 logMAR. Analyzing PERG data, we observed significant differences in the P50-N95 amplitudes of the amblyopic group compared with the NE group (*p* < 0.0001–amblyopic eye vs. NE; *p* = 0.039–fellow eye vs. NE).

**Discussion:**

These findings suggest that amblyopic patients may also present other impairments beyond the visual cortex. PERGs seem to be an important complementary examination in the diagnosis of other impairments in amblyopia.

## Introduction

Pattern electroretinogram (PERG) is a noninvasive technique that provides a retinal biopotential evoked by a black and white checkerboard pattern temporally modulated in high contrast [1]. PERG can be a highly sensitive indicator of changes in the macular area, caused by dysfunction of the optic system, bipolar cells, photoreceptors, and/or ganglion cells [2]. Clinically, PERG can also be used as a tool to detect primary ganglion cell disease, optic neuropathies, and/or glaucoma.

Amblyopia is generally a unilateral disorder, with a worldwide prevalence between 3 and 6% [3, 4]. This disorder is defined by a difference of at least two lines of visual acuity between the eyes with the best-corrected visual acuity, a decrease in contrast sensitivity, and a decrease in stereopsis [4]. The most frequent amblyopic factors are anisometropia (a difference of refractive error between the eyes), strabismus (misalignment of the eyes), or a congenital occlusion (ptosis) [4]. The main theorized explanation for amblyopia relates to the different image perception seen by each eye, leading to a cortical mismatch that will confuse image interpretation by the brain. This process will probably cause an abnormal visual cortex inhibition, concerning the amblyopic eye. Due to this imbalance, the non-amblyopic eye will take control of the visual system, suppressing the eye with blurred vision [5, 6]. It is believed that amblyopia has a central origin in the brain, specifically in cortical area V1 [7–9]. Previous research has demonstrated that there are not only functional but also anatomic architecture changes in the V1 region [10, 11]. Even though the primary locus of dysfunction in amblyopia is commonly believed to occur in the primary visual cortex, some studies suggest that abnormalities may also be present in extrastriate and secondary specialized cortical areas. These findings suggest that the visual deficits associated with amblyopia extend beyond V1 and involve higher-level visual processing areas. Conversely, neurophysiologic studies in amblyopic monkeys have shown that the neuronal acuity loss in V1 may be not enough to reflect losses in measured visual acuity [12, 13]. In addition to post-chiasmatic changes, there are authors who argue that some pre-chiasmatic changes may also be present, and that these impairments may not be secondary to the post-chiasmatic dysfunction. These theories are supported by functional MRI findings such as thalamic dysfunction in amblyopic patients and the reduced quality of the signal emitted by the retinal cells of the amblyopic eye when they reach the cortex [9, 14].

Recent research defends a different theory addressing the functionality of retinal cells in the amblyopic eye. A few studies, almost all in children, found abnormal PERG responses in amblyopic patients, although they did not report uniform abnormalities either in amplitudes, latencies, or simultaneous changes [6, 7, 10–12]. Some authors like Sokol et al. (1979) already observed that PERG had greater sensitivity in finding changes than flash electroretinograms in amblyopic patients [15]. On the other hand, contradicting findings were reported by some authors like Hess et al. (2009) and Gottlob et al. (1987), who found no significant changes in PERG amplitude and/or latency in amblyopic eyes [9, 16].

PERG is a suitable test for making the study of amblyopic patients more complete, by enabling evaluation and comparison responses from the normal and the amblyopic eye [6, 9, 11]. Considering that there are very few recent studies using PERG in amblyopic adults [17], with contradicting results, it seems relevant to study this topic in greater depth. Therefore, we aimed to evaluate the PERG retinal responses of adult amblyopic eyes compared with normal eyes, using the latest guidelines, attempting to better understand the role of PERG in the diagnosis of amblyopia.

## Materials and methods

Twenty-four adult volunteers, twelve amblyopes, and twelve subjects with normal eye (NE) were included in this study. The NE subjects were mostly students recruited in University of Beira Interior, in Covilhã, Portugal, and the amblyope subjects were found through visual screening of over one thousand factory workers in the surrounding region.

None of the subjects with NE presented amblyopia or strabismus or other visual problems. As criteria for inclusion in the amblyopic group, we considered difference in spherical equivalent refractive error between the eyes superior or equal to 1.50D; ocular deviations (strabismus and microstrabismus); difference in visual acuity (VA) superior or equal to two lines between the eyes [18, 19]. All subjects of the amblyopic group already knew they have a difference of VA between the eyes.

A complete optometric screening was performed in all subjects to discard ocular diseases, through retinography, optical coherence tomography, corneal topography, axial length (AL) measurement, and slit-lamp examination of the anterior and posterior segments. (All volunteers did not show changes in the examinations performed.) All the subjects with a history of systemic or ocular disease or taking any medication were excluded.

A cover test measurement from far and near was also performed to assess ocular deviations, and the visual acuity (VA) measurement from far and near was performed using a logMAR scale. Both tests was performed at 4 m and 40 cm, respectively, in accordance with the recommendations of Elliot D (2007) [20]. According to the guidelines from previous studies, the importance of using the best compensation, which allows achieving the best VA, is an important parameter to consider in PERG performance, as it will allow more reliable test results [2]. All the volunteers used the appropriate compensation for the distance at which the test was been performed. PERG was performed using a Retimax system for ocular electrophysiology, and the technique was mostly based on ISCEV guidelines [2]. PERG was performed at 30 cm with a reversal checkboard of black and white squares with 2.9° degrees (Model Display 42PT353-ZA). Screen parameters: contrast of 99.73% (maximum luminance 100.65 ± 0.95 cd/m^2^ and minimum luminance 0.05 ± 0.05 cd/m^2^) and 2 Hz frequency. The procedure was conducted binocularly under dim room lighting conditions, with non-dilated pupils. In the case of subjects with strabismus, the procedure was carried out exclusively in a monocular fashion, with the non-tested eye patched, in accordance with the ISCEV guidelines. This approach ensures compliance with the standardized protocols recommended for individuals with strabismus during PERG measurements. We used HK-loop electrodes (electrodes tolerance resistance 5000 $\Omega$)—ground electrode was positioned on the forehead and reference electrodes (10 mm silver plated disc electrodes) were placed on the skin near the ipsilateral outer canthus of each eye, in accordance with ISCEV guidelines. The amplifier of the Retimax equipment has two channels for simultaneous recording, with inputs for active, reference, and ground electrodes. The notch filter was turned on. Regarding the filter bandwidth, the specifications used were high pass 1 Hz and low pass 30 Hz. PERG P50-N95 amplitude, and P50 and N95 latencies were evaluated [21].

During the acquisition of PERG data, the quality of stimulus fixation was visually controlled by the examiner. During data collection, an automatic signal rejection was used (amplitude-based), to ensure the best signal quality. PERG latency and amplitude determination were carried out blindly, by another member of the research group.

All participants gave their written informed consent to participate in the study. The study was approved by the Ethics Committee of the University of Beira Interior and was conducted in accordance with the principles of the Declaration of Helsinki.

All data were analyzed using the SPSS Statistics 27 software. When comparing data from the amblyopic eye with data from the fellow eye we used a non-parametric Wilcoxon test because the assumption of normality was not verified. To compare results between the amblyopic group and the group with subjects with NE we used a non-parametric Mann–Whitney test because the assumption of normality was not verified. Bonferroni correction was applied whenever multiple comparison were made. For the statistical evaluation, we used a significance value of *p* < 0.05. To determine the degree of correlation between best-corrected visual acuity and PERG response, Pearson’s correlation was used. For statistical evaluation, were considered *p* values between − 1 e 1.

## Results

The control group (NE) was composed of twelve subjects with normal binocular vision (6 females and 6 males), aged between 22 and 54 (mean 35.58 ± 12.85 years) with spherical equivalent refractive error between 0 and − 4.00 diopters (mean − 0.95 ± 1.65 diopters). In this group, the mean of axial length measured was 23.77 ± 0.88 mm.

The amblyopia group was composed of twelve subjects with amblyopia (10 females and 2 males) in which we found 4 cases with anisometropia, 6 with strabismus, and 2 with mixed causes (anisometropia and strabismus). The amblyopic subjects were aged between 20 and 59 years (mean 43.42 ± 12.72 years old), with spherical equivalent refractive error between -10.25 and + 6.00 diopters (mean -2.03 ± 4.29 diopters). In this group, the mean of axial length measured was 24.23 ± 1.74 mm. The difference between the axial length in the two groups was 0.47 mm, which corresponds to an approximate variation of 2%.

When comparing the NE group with the amblyopic group in terms of age there were no significant differences between the two groups (Mann–Whitney = − 1.504; *p* = 0.143). No significant differences were found regarding gender (Mann–Whitney = − 1.319; *p* = 0.187).

Table 1 presents the average values obtained for optometric parameters and PERG examination in the two groups: the NE and amblyopic groups. Comparing the PERG P50-N95 amplitude, P50 latency, and N95 latency between the two eyes of the NE group, no significant differences were found. The eye chosen as the control in this comparison was selected randomly. Additionally, there were no significant differences in axial length (AL) between the amblyopic group and the NE group (Mann–Whitney = − 0.866; *p* = 0.410).

**Table 1:** Clinical data and PERG results in NE group, amblyopic eye, and fellow eye

	NE, *N* = 12 (Mean ± SD)*	Amblyopes, *N* = 12	
		Amblyopic eye (Mean ± SD)* (%)**	Fellow eye (Mean ± SD)* (%)**
Refractive error (SE)	− 0.95 ± 1.65	− 2.03 ± 4.29	− 0.08 ± 3.01
VA far (logMAR)	0.00 ± 0.00	0.41 ± 0.20	− 0.02 ± 0.14
VA near (logMAR)	0.00 ± 0.00	0.37 ± 0.24	0.06 ± 0.11
PERG P50-N95 amplitude (μV)	20.90 ± 7.12	14.41 ± 3.71 (47.43)	15.76 ± 4.20 (42.50)
PERG P50 latency (ms)	48.96 ± 1.86	48.48 ± 3.10 (1.11)	48.27 ± 2.51 (0.67)
PERG N95 latency (ms)	101.20 ± 7.88	103.26 ± 7.86 (5.93)	103.75 ± 10.68 (6.43)

^*^Mean: Mean value of the results from the 12 volunteers; *SD*: Standard deviation of the results from the 12 volunteers

^**^%: Difference percentage between mean values obtained in normal eyes with amblyopic eye and normal eyes with fellow eye

**Fig. 1:**
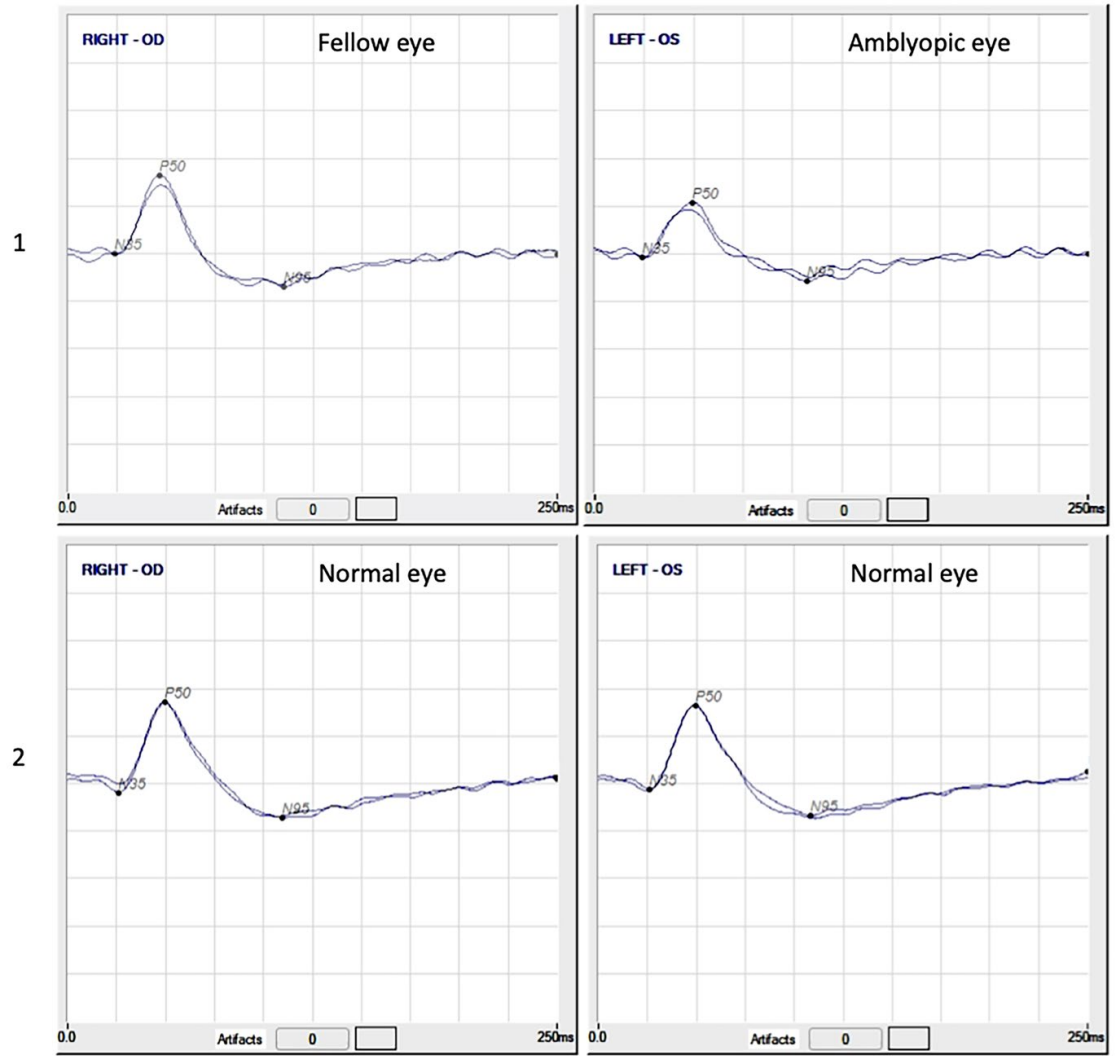
PERG–Samples of two reproducible trials per volunteer. 1. Samples of an amblyopic volunteer (amblyopic eye (right); fellow eye (left)) & 2. Samples of a normal vision volunteer (normal right and left eyes). Scales: 9 µV/division and 25 ms/division. 1. Mean values right eye: P50-N95 amp. = 22.15 µV, P50 lat. = 46.88 ms; Mean values left eye: P50-N95 amp. = 15.39 µV, P50 lat. = 48.58 ms; 2. Mean values right eye: P50-N95 amp. = 21.89 µV, P50 lat. = 49.80 ms; Mean values left eye: P50-N95 amp. = 21.35 µV, P50 lat. = 49.07 ms

Figure 1 shows samples of records from an amblyopic volunteer and a volunteer with normal eye.

**Fig. 2:**
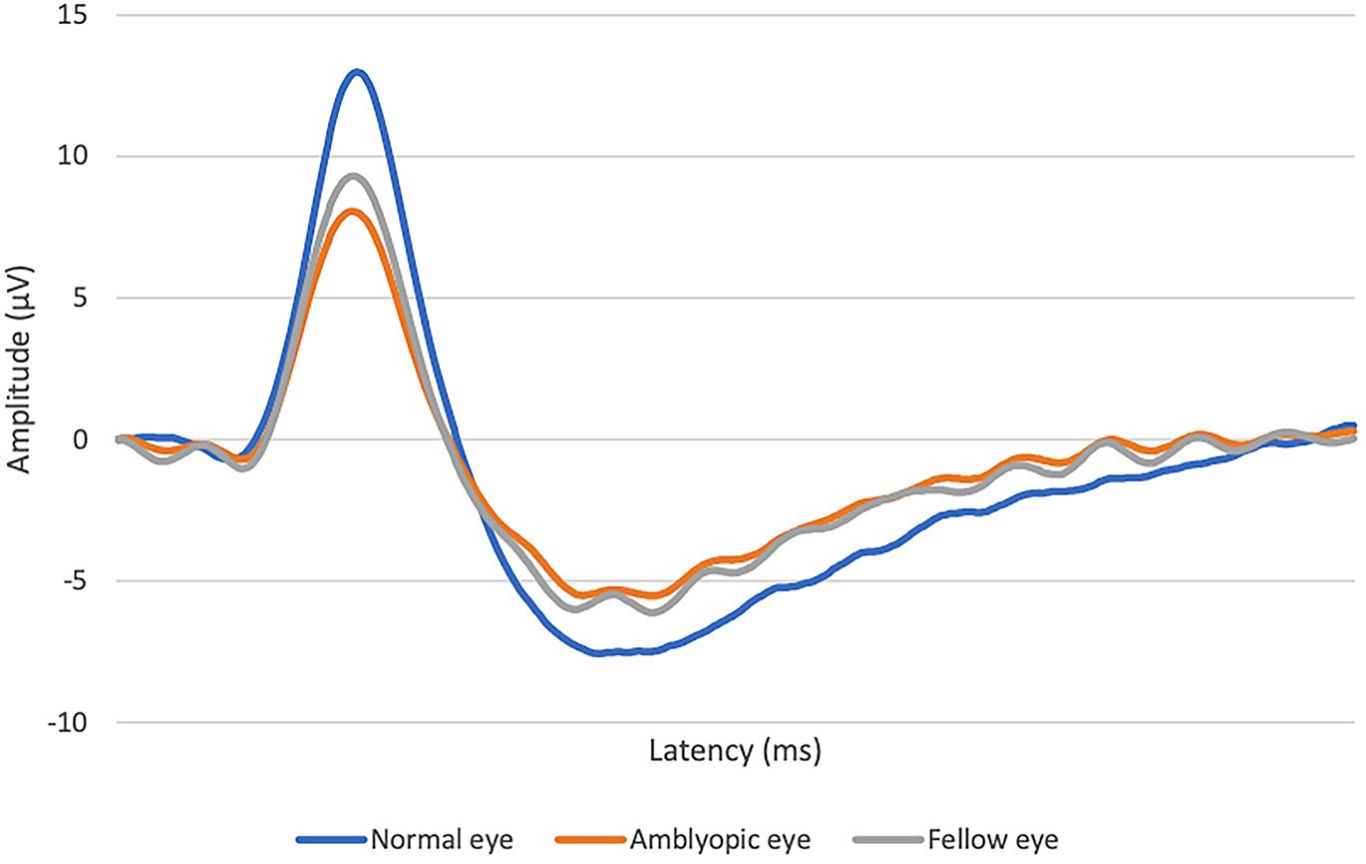
Mean PERG response in normal eyes, amblyopic eyes, and fellow eyes

Figure 2 shows the behavior in PERG response in normal eyes, amblyopic eyes, and fellow eyes.

Analysis of PERG data obtained from amblyopic subjects, showed no significant differences when the amblyopic eye was compared with the fellow eye in all parameters: P50-N95 amplitude (Wilcoxon = − 1.334; *p* = 0.182), P50 latency (Wilcoxon = − 0.549; *p* = 0.583) and N95 latency (Wilcoxon = 0.235; *p* = 0.814).

However, significant differences in P50-N95 amplitude were observed when comparing PERG results between the amblyopic subjects and the NE (normal eyes) group, as indicated in Table 2.

**Table 2:** PERG comparisons between the NE and amblyopic group

PERG	*p* value*	
	NE versus amblyopic eye	NE versus fellow eye
Amplitude P50-N95 (μV)	**0.001**	**0.039**
Latency P50 (ms)	0.630	0.198
Latency N95 (ms)	0.887	0.755

^*^Mann–Whitney U test

The p-values in bold correspond to statistically significant values (p<0,05). Significant differences were found in PERG P50-N95 amplitude (tendency to decrease amplitude in the amblyopic group subjects) when comparing the NE group with the amblyopic eye and the fellow eye

Furthermore, significant differences were also found in PERG P50-N95 amplitude (tendency to decrease amplitude in the amblyopic group subjects) when comparing the NE group with the amblyopic eye and the fellow eye, respectively. No differences were found in PERG latency parameters (P50 or N95).

Pearson’s correlation was calculated between the VA of the amblyopic eyes and the P50-N95 amplitude (Pearson correlation = − 0.610; *p* = 0.035), P50 latency (Pearson correlation = 0.684; *p* = 0.014), and N95 latency (Pearson correlation = 0.665; *p* = 0.018). All correlation values are weak, and we found no correlation between the VA and PERG responses.

## Discussion

We found no differences in PERG responses between the amblyopic eye and the fellow eye in amblyopic patients, but when comparing PERG responses of amblyopic subjects with PERG responses from the normal vision group (NE), we found a significant decrease in P50-N95 amplitude, both in the amblyopic eye and in the fellow eye in the amblyopic patients. These differences were also seen when different subgroups of amblyopic subjects were compared with NE subjects. No significant differences were found in PERG latencies. These results seem to support the theory that amblyopia may not be an exclusively cortical-dependent condition.

The P50-N95 amplitude response consists of the maximum amplitude intensity of the signal sent when the macular cells are activated by a stimulus. PERG latency consists of the time necessary for the macular cells to reach the maximum intensity of response to a stimulus [22]. These components are related to each other, and also to the brightness of the background–in general, the amplitude response increases significantly after light adaptation [23].

Some authors propose that latencies of PERG responses are mostly constant and rarely change in different diseases of the retina and optic nerve [1, 15]. This may be the basis for the theory that amblyopia does not lie in the path the signal takes (PERG latency response without significant changes), but in the reduced intensity with which retinal cells send the signal. Sokol et al. (1979) put forward three possibilities to explain the decrease in PERG amplitude response in amblyopic subjects: i) the retinal image was blurred, affecting the contrast level between checks, thereby reducing the amplitude response; ii) there is an unstable fixation by the amblyopic eye, thus eye movements may affect amplitude; and iii) the possibility that there is a dysfunction in the mechanism of photopic retinal cells in the amblyopic eye (such as occurs in retinitis pigmentosa) [15]. Kiorpes et al. (2016) suggest that in amblyopia the initial information that arrives at the visual cortex from the amblyopic eye is weak [14]. On the other hand, Hess et al. (2009) claim that retinal cells and some layers of the lateral geniculate nucleus (LGN) are abnormal in the amblyopic eye, with the presence of a cortical deficit with reduced activations in V1 and extrastriate cortex, with no effect from the retinal cell response [9]. No definite theory has been defined in order to explain abnormalities in PERG response in amblyopia.

### Amblyopic eye versus fellow eye

Our findings suggest that there are no significant differences in the results obtained in PERG response when comparing the amblyopic eye with the fellow eye (amplitude and /or latency). These results are in line with Parisi et al. (2010), Tugcu et al. (2013), and Esposito Veneruso et al. (2014) [7, 24, 25]. Parisi et al. (2010) justify their findings of no significant differences in PERG responses between the amblyopic and fellow eyes by suggesting that there is only an abnormal cortical response in amblyopic subjects and thus the eye itself is normal [24].

No differences in PERG latencies, particularly, were found. Some authors have noted that the latencies of PERG responses are mostly constant and rarely change in different eye diseases [1, 15]. Gottlob et al. (1987), Hess et al. (1985), and Hess et al. (2009) found similar results and defended that retinal function in amblyopic eyes is similar to the retinal function in subjects with NE, suggesting that the changes with amblyopia primarily occur in the brain, specifically at the level of the primary visual cortex [9, 16, 26].

### Amblyopic eye versus NE

The theory relating amblyopia origin to the cortex does not explain our findings in the amplitude parameter. No significant differences were found in latency parameters. The significant differences in amplitude found when comparing the results of the amblyopic eye group with the group of NE subjects (lower amplitudes in amblyopic PERG response) are in agreement with the work of Heravian et al. (2011) who developed his study in amblyopic adults, using volunteers with NE of the same age as the group of amblyopes [17]. These researchers suggested that the apparent retinal defect may be related to the malfunction of the visual cortex [17]. This theory is supported by the work of Yin et al. (1994), who also state that a retinal defect is associated with probable cortical defects, although the retinal defects have smaller depth [17, 27]. Feller et al. (1996) further explained this theory, believing that the information corresponding to the eye layers at thalamic level depends on retinal waves of spontaneous activity that are generated on nicotinic acetylcholine receptor activation. Although this activity is not required for the development of ganglion cells, it is necessary for the anatomical and functional development of the post-retinal visual system [28]. We understand that it is possible that retinal impulses can affect visual system development.

### Amblyogenic factors

The conclusions about the results of these subdivisions by amblyogenic factor were limited by the small size of the sample. It is important to obtain more amblyopes in order to be able to make this subdivision of the amblyopic group, because with our sample we have unequal and reduced subgroups.

Regarding the fellow eye comparisons, our results follow the theory defended by Huang et al. (2011), who observed that the imbalance between the eyes with anisometropic amblyopia may lead to functional impairment not only of the amblyopic eye, but also affects the visual network of the fellow eye and the interaction between the two eyes [29]. These deficits could be related to abnormal direct and indirect interocular inhibition between the fellow eye and the amblyopic eye, affecting binocular balance and thus monocular visual performance [29]. Other authors like Harrad et al. (1992) already defended this theory, by suggesting that the binocular dysfunction was not just a consequence of the monocular loss but also depended on the etiology of the amblyopia and the spatial frequency of the stimulus presented to the eye [30].

As was verified in the Heravian et al. (2011) work, we expected that there would be a strong correlation between VA and PERG amplitude responses; however, in our sample, we only saw a trend suggesting that with reduced VA, there is a tendency to decrease the amplitude response [17]. It could be hypothesized that AL could verify this difference. R. Hidajat et al. (2003) found that for 1 mm of AL difference, the amplitude response decreases 11.6%, in normal volunteers, and E. Grudzinska et al. (2021) defends that the amplitude response decreases 7.3%, in myopes, also for each 1 mm of difference [31, 32]. In our study, there were no significant differences in axial length between the normal eye group and the amblyopic group (*p* = 0.410), with an AL of only 0.47 mm. Based on these findings, one could expect a decrease in amplitude up to approximately half the result found by Hidajat et al. However, the observed difference in amplitude between our two groups is much greater, suggesting that the decrease in amplitude in amblyopic eyes cannot be solely attributed to the difference in axial length.

Finally, our results show that there is a decrease of amplitude response of the retinal cells in the amblyopic eye and the fellow eye when compared with eyes of the normal vision control group. We also observed that the amblyogenic factor does not seem to be relevant in terms of the response of retinal cells. Our results support the findings of more recent studies, which underlie the hypothesis that there is an abnormal component in the more peripheral neurological vision (like LGN, optic nerve, and/or retina), where changes in the amplitude response of retinal cells may also be observed in amblyopic eyes [33, 34]. A limitation of our study may be the difficult of monitoring fixation loss, although the researcher was always present encouraging the subjects to look at the right spot. Nevertheless, in order to further characterize our findings, studies with a larger sample size are needed.

Our findings show that PERG may prove to be an important additional test in the diagnosis of amblyopia, contrary to what was believed a few years ago, and also seems to help to clarify the conflicting findings on previous literature.

## Data Availability

Data available for request.
